# Minimising disability and falls in older people through a post-hospital exercise program: a protocol for a randomised controlled trial and economic evaluation

**DOI:** 10.1186/1471-2318-9-8

**Published:** 2009-02-26

**Authors:** Catherine Sherrington, Stephen R Lord, Constance M Vogler, Jacqueline CT Close, Kirsten Howard, Catherine M Dean, Lindy Clemson, Elizabeth Barraclough, Elisabeth Ramsay, Sandra D O'Rourke, Robert G Cumming

**Affiliations:** 1 Musculoskeletal Division, The George Institute for International Health, The University of Sydney, PO Box M201, Missenden Rd, Sydney, NSW 2050, Australia; 2 Prince of Wales Medical Research Institute, UNSW, PO Box 82, St Pauls, NSW 2031, Sydney, Australia; 3 Department of Aged Care and Rehabilitation, Royal North Shore Hospital, St Leonards, Sydney, Australia; 4 Department of Geriatric Medicine, Prince of Wales Hospital, Sydney, Australia; 5 School of Public Health, The University of Sydney, Sydney, Australia; 6 Discipline of Physiotherapy, Faculty of Health Sciences, The University of Sydney, Sydney, Australia; 7 Discipline of Occupational Therapy, Faculty of Health Sciences, The University of Sydney, Sydney, Australia; 8 Centre for Education and Research on Ageing, Concord Hospital, Sydney, Australia

## Abstract

**Background:**

Disability and falls are particularly common among older people who have recently been hospitalised. There is evidence that disability severity and fall rates can be reduced by well-designed exercise interventions. However, the potential for exercise to have these benefits in older people who have spent time in hospital has not been established.

This randomised controlled trial will investigate the effects of a home-based exercise program on disability and falls among people who have had recent hospital stays. The cost-effectiveness of the exercise program from the health and community service provider's perspective will be established. In addition, predictors for adherence with the exercise program will be determined.

**Methods and design:**

Three hundred and fifty older people who have recently had hospital stays will participate in the study. Participants will have no medical contraindications to exercise and will be cognitively and physically able to complete the assessments and exercise program.

The primary outcome measures will be mobility-related disability (measured with 12 monthly questionnaires and the Short Physical Performance Battery) and falls (measured with 12 monthly calendars). Secondary measures will be tests of risk of falling, additional measures of mobility, strength and flexibility, quality of life, fall-related self efficacy, health-system and community-service contact, assistance from others, difficulty with daily tasks, physical activity levels and adverse events.

After discharge from hospital and completion of all hospital-related treatments, participants will be randomly allocated to an intervention group or usual-care control group. For the intervention group, an individualised home exercise program will be established and progressed during ten home visits from a physiotherapist. Participants will be asked to exercise at home up to 6 times per week for the 12-month study period.

**Discussion:**

The study will determine the impact of this exercise intervention on mobility-related disability and falls in older people who have been in hospital as well as cost-effectiveness and predictors of adherence to the program. Thus, the results will have direct implications for the design and implementation of interventions for this high-risk group of older people.

**Trial Registration:**

The protocol for this study is registered with the Australian New Zealand Clinical Trials Registry ACTRN12607000563460.

## Background

The development and implementation of effective strategies to minimize disability and falls among older people is an urgent public health challenge due to the increasing proportion of older people in the global population. Around 500 million people worldwide are aged 65 and older and this number is predicted to increase to 1 billion by 2030 which will represent one eighth of the global population. Developing countries are predicted to have a 140 percent increase in older people by 2030.[1]

In the World Health Organization's International Classification of Functioning, Disability and Health (ICF).[2], disability is an umbrella term for impairments (in body structure or physiological function), activity limitations (difficulties an individual may have in executing activities, tasks or actions) and participation restrictions (difficulties with involvement in life situations). Mobility is a domain of the ICF which poses difficulties for many older people. Australian data indicate that close to one quarter of people aged over 65 years report a severe or profound limitation of core activities (self-care, mobility or communication) and that 84% of these people report mobility limitations.[3] There is increasing evidence that this mobility-related disability in older people can be reduced by well-designed exercise programs. The LIFE-P randomized trial found that mobility-related disability (measured on the Short Physical Performance Scale) could be reduced with a combination of supervised and home-based exercises.[4]

At least 30% of people aged 65 and over fall at least once each year.[5] Fall rates are even higher in people who have been in hospital. Fourteen percent of people fall in the first month after discharge from hospital.[6] Systematic reviews of falls prevention strategies in older people have found that falls can be prevented by certain exercise programs.[7] In particular, ongoing exercise which targets balance can prevent up to 40% of falls.[8]

Admissions to hospital in older people are strongly associated with a decreased level of functioning and subsequent falls. A prospective study found that people without pre-existing disability are many times more likely to develop a disability within a month of a hospital stay than those who have not had hospital admission (adjusted hazard ratio = 59.8, 95% CI 46.6 to 76.8).[9] Thus, people after recent hospital stays are clearly a group who may benefit from exercise programs designed to lessen or prevent this disability. In one of the few trials of exercise in a post-hospital population [10], intensive training (three sessions a week for three months) improved physical performance significantly and between-group differences were maintained two years later.[11] We have found that home-based exercise programs which include balance and strength training can improve performance on physical tests which are predictive of falls.[12] However, it is not clear whether fall prevention exercise programs can be effectively implemented in people who have recently been hospitalized. A previous study found no effect of a seated strengthening program on fall rates in people who had been in hospital (relative risk = 0.96, 95% CI 0.67–1.36).[13]

While the increased risk of falls and disability after hospital discharge decreases with time for many individuals, for many others this risk will not return to pre-admission levels without specific intervention. Evidence suggests that a substantial number of people who have been recently hospitalized will be at an increased likelihood of falls and disability and their consequences: fractures, fear of falling, activity restriction, further decline in physical functioning and a move to institutional care.[14] Therefore, we have designed a post-hospital home-based intervention which aims to minimize disability and falls in the high-risk post-hospital population and will evaluate this intervention in a randomized controlled trial. In order for an intervention to be implemented, an understanding of its cost-effectiveness as well as its effectiveness is required. Thus, we will also conduct an economic analysis.

Adherence to exercise programs is a major issue in public health research and clinical practice. Predictors of adherence to exercise programs in older people at risk of falls and disability are not well understood [15] but useful descriptive work has been undertaken.[16] There is some evidence that longer term adherence to exercise may be better with home-based rather than center-based programs for older adults.[17] Within our randomized trial we will also establish predictors for adherence to a home-based exercise program in the post-hospital population.

To date, there is little evidence that falls and/or mobility-related disability can be minimized in older people recently discharged from hospital. This randomised controlled trial aims to evaluate the effect of an intervention primarily based on the Weight-bearing Exercise for Better Balance (WEBB) exercise program in preventing falls and minimizing disability in people who have recently been in hospital. The trial will also establish predictors of adoption and adherence to the exercise program and enable an analysis of cost effectiveness.

## Methods and design

### Design

**Figure 1:**
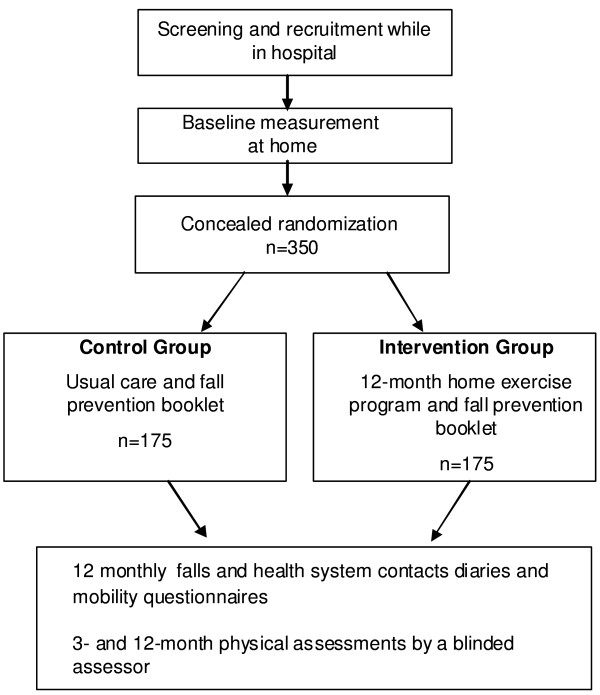
**Flow of participants through the trial**.

A randomized controlled trial will be conducted among approximately 350 participants recently discharged from hospital. Figure 1 gives an overview of the study design.

### Participants

Participants will be consenting people aged 60 years and over who have been admitted to and subsequently discharged from participating public hospital wards in Sydney, Australia.

People will be ineligible to participate in the trial if they: reside in a high-care residential facility, have a cognitive impairment (a Folstein Mini-Mental State Examination [18] score of less than 24 after any acute confusional state has resolved), have insufficient English language skills to understand the assessment and/or intervention procedures (unless they have a carer who can assist), are unable to walk more than one meter despite assistance from a walking aid and/or another person, have a progressive neurological disease (e.g. Parkinson's disease) or suffer from any medical condition precluding exercise (e.g. unstable cardiac disease).

Interest in study participation will be sought during the hospital stay but assessment and randomization will occur after discharge from hospital and after any associated home rehabilitation organized as a result of the hospital admission.

The University of Sydney Human Research Ethics Committee (HREC) approved the study protocol (HREC Number 12-2006/9682) as have ethics committees at the participating hospitals.

### Randomization

After completion of the baseline assessment, participants will be formally entered into the study and randomized to intervention or control groups. Randomization will be stratified by hospital site and falls history (0–1 versus 2+ falls in the previous 12 months) using a computer-generated random number schedule with variable block sizes of 2–6. Randomization will be performed centrally by an investigator not involved in recruitment or assessments.

### Intervention

Prior to commencement of the intervention, each participant's medical practitioner will be contacted to ensure that no new medical issues have arisen and that he/she supports the participant's involvement in the study.

Each participant in the intervention group will receive 10 home visits from a physiotherapist (physical therapist) in the 12-month study period. Each visit will last 40 – 60 minutes as required, and the participants will then be requested to undertake a 20 – 30 minute program of lower limb balance and strengthening exercises up to 6 times per week at home for 12 months.

The exercise intervention program will be based on the Weight-bearing Exercise for Better Balance (WEBB) program developed by two of the authors (CS and CMD) and colleagues. [19] Exercises will be primarily conducted while standing. We have previously found additional mobility benefits of standing exercise over seated exercise without adverse effects.[12,20-22] In addition, our recent meta-analysis found that exercise which challenges balance to a greater extent has greater effects on falls prevention.[8].

The exercises will aim to reduce falls and enhance balance and mobility by targeting postural control and muscle strength. After an assessment of each participant's physical abilities, study physiotherapists will individually prescribe the number and choice of exercises, number of repetitions to be completed and level of difficulty of prescribed exercises. Exercises which primarily target postural control (balance) include standing with a narrower base (aiming for tandem or single leg stance), forwards and sideways stepping/walking, and graded reaching activities in standing. The lower limb extensor muscle groups, which act to prevent collapse of the lower limb (hip and knee extensors and ankle plantarflexors) will be targeted with exercises which aim to enhance muscle strength and control. Strengthening exercises will include sit-to-stand, forward and lateral step-ups onto a small block, and heel raises in standing. Resistance for strengthening exercises will be added to these tasks by the use of weight-belts worn around the waist or weighted vests. The appropriateness of using added resistance for each individual participant will be assessed by the study physiotherapist in consultation with medical practitioners. The use of upper limbs to support the body while exercising will be minimized but will be encouraged where necessary to ensure safety.

As this study involves the prescription of home-based exercise to a high risk population, safety while exercising will be a prime consideration when exercises are prescribed. Participants will be instructed how to perform exercises with stable supports (such as a table) located nearby to hold onto if needed. Where appropriate, family members and/or carers will be encouraged to assist with supervision of the exercise program. If a participant becomes unwell or has another admission to hospital, the program will be resumed when the participant and the relevant professionals deem him/her well enough to participate again.

The optimal intensity and type of exercises for each individual participant will be regularly assessed and adjusted by the study physiotherapists to ensure that the intervention remains challenging as a participant's performance improves. A manual for physiotherapists has been developed to assist in the use of this program and is available from the authors upon request.

Participants will be provided with a booklet containing safety precautions, instructions and photographs of exercises for use in exercise sessions at home. In addition, they will be provided with a logbook for recording exercises completed and effects of exercise (e.g. muscle soreness). The Physical Activity Stage of Change model [23] will be used by the study physiotherapists to guide their approach to encouraging ongoing home-based exercise participation.

The control group will receive their usual care from their medical practitioners and community services so will not be disadvantaged by being in the study.

Participants in both groups will also receive an education booklet about fall prevention.

### Outcomes

#### Data collection

Data will be collected from medical records, postal questionnaires and calendars, and physical assessments. Information on medical history, diagnoses, medication and unintentional weight loss will be collected from medical records while participants are still in hospital.

All participants will undergo three home-based assessments. The baseline assessment will be conducted prior to randomization. Further assessments will be conducted three and 12 months after randomization. Assessments will be conducted by physiotherapists or trained research assistants and will involve questionnaires and physical performance assessments. Each assessment will take about one and a half hours to complete. All assessors will be unaware of group allocation of participants they are assessing. Participants will be instructed not to inform the assessors of their intervention status, and all exercise equipment will be removed prior to assessment.

All participants will receive 12 calendars and questionnaires at the time of the baseline assessment. Participants will be asked to record falls and use of health and community services on the calendars and complete questionnaires about mobility-related disability (monthly) and quality of life (3-monthly) and return completed calendars in pre-paid envelopes to the research centre each month. Participants who do not return calendars or questionnaires will be telephoned to ask for the information. Participants who report falling will also be telephoned to seek more information about the circumstances and consequences of the fall. Staff who receive calendars and questionnaires, make follow up phone calls and enter data will be unaware of group allocation.

#### Primary outcome measures

Mobility-related disability and falls are the primary outcome measures in this trial.

Mobility-related disability will be measured monthly with a self-reported test and at 3- and 12-months after randomization using a performance-based test. The monthly mobility measure will involve a self-reported questionnaire about difficulty in mobility tasks using the physical and movement items suggested by Haley et al.[24]

The performance-based mobility measure will be the lower extremity Summary Performance Score version [25] of the Short Physical Performance Battery.[26] This battery gives a composite score based on timed performance of three mobility tasks: the ability to stand for up to 10 sec with feet in different positions (together side by side, semi-tandem and tandem), 4-meter walk and time to rise from a chair five times.

Falls will be measured monthly using the calendars as outlined above. A fall will be defined according to the Kellogg definition [27] as an incident in which the body unintentionally comes to rest on the ground or other lower level which is not as a result of a violent blow, loss of consciousness, sudden onset of paralysis as in a stroke or an epileptic seizure.

#### Secondary outcome measures

Secondary outcome measures will be risk of falling, additional measures of mobility, strength and flexibility, quality of life, fall-related self efficacy, health-system and community-service contact, assistance from others, difficulty with daily tasks, physical activity levels and adverse events. These aim to enhance understanding of the effects of exercise on multiple factors that contribute to an older person's abilities and quality of life, and to enable economic analyses to be conducted. These outcome measures are described below.

Fall risk will be assessed using the *Physiological Profile Assessment *[28] which includes measures of knee extension strength, postural sway, lower limb proprioception, reaction time and visual contrast sensitivity. *Choice Stepping Reaction Time *has been found to be a composite measure of risk of falling when assessed with an electronic device.[29] We will measure this in a modified manner with a portable mat. A mat with four white squares will be placed in front of the standing subjects, and they will be asked to make a standard number of steps with either foot to a particular square and back again, using a standardized script. The time for the entire routine will be recorded in seconds.

Additional measures of mobility to further explore posited effects of the intervention will be assessed using the *Maximal Balance Range *and *Coordinated Stability Tests*.[30] The Maximal Balance Range test assesses subjects' ability to lean as far forward and backwards as possible. The Coordinated Stability requires subjects to adjust their body position in a steady and coordinated manner when near the limits of their base of support. *Grip strength *will be assessed using a portable dynamometer (JAMAR Hydraulic Hand Dynamometer manufactured by Sammons Prestons). *Ankle flexibility *will be measured in a torque-controlled manner using a modification of the Lidcombe template procedure [31] in which the person is sitting rather than lying and an inclinometer is used to assess changes in ankle angle. In addition, the effects of the intervention on the individual components of the Short Physical Performance Battery and single leg stance time will be assessed.

Quality of life will be assessed using the *SF 12*™*Version 2 *at baseline, 3 and 12 months and each 3 months using the postal *EQ-5D*.[32]

Fall-related self-efficacy will be assessed using the *Falls Efficacy Scale-International *[33] in which level of concern about falling when carrying out a range of activities is rated on a 4-point scale.

*Health and community service contact *will be assessed using calendars as described above. Participants will be asked about the need for *assistance from others*.

*The level of difficulty with daily tasks *will be assessed using the personal care and instrumental items suggested by Haley at al.[24]

*Physical activity levels *will be assessed with the Physical Activity Questionnaire for older people (developed by SRL).

Exercise *program adherence rates *and *minor adverse effects *(eg. muscle pain not limiting daily activities) of the exercise program will be monitored using exercise diaries kept by participants. Participants will be advised to telephone study staff if they experience any *major adverse effects *(e.g. chest pain, muscle soreness lasting for more than 48 hours and interfering with daily activities or requiring medical attention).

*Exercise self-efficacy *will also be assessed and participants will be asked to *identify reasons for exercise adherence *at the follow-up interviews.

### Statistical analysis

The number of falls per person-year will be analyzed using negative binomial regression to estimate the difference in fall rates between the two groups.[34] The proportion of fallers in the two groups will also be compared using the relative risk statistic. Longitudinal mixed models will be used to assess the effect of group allocation on the continuously-scored primary and secondary outcome measures. Logistic regression models will be used to compare groups on dichotomous outcome measures. An intention-to-treat approach will be used for all analyses. Analyses will be conducted using the SPSS and STATA software packages. Predictors of exercise adherence will be established using multivariate modeling techniques.

### Economic analysis

Data will be collected regarding costs of exercise program delivery (including staff costs, training, capital costs and consumables), inpatient hospital admissions, emergency department presentations and other health and community service contact (from the monthly calendars). Incremental cost-effectiveness ratios will be calculated in terms of a) the incremental cost per participant achieving a significant increase in physical functioning (half of one point on the Short Physical Performance Battery which is considered to be a small meaningful change [35]), b) the incremental cost per fall prevented, and c) the incremental cost per quality-adjusted life year (QALY) gained in the exercise group compared with the control group. Results will be plotted on a cost-effectiveness plane. Bootstrapping will be used to estimate a distribution around costs and health outcomes, and to estimate the confidence intervals around the incremental cost-effectiveness ratio. One-way sensitivity analysis will be conducted around key variables, and a probabilistic sensitivity analysis will be conducted to estimate the joint uncertainty in all parameters; a cost-effectiveness acceptability curve (CEAC) will be plotted. A CEAC provides information about the probability that an intervention is cost-effective, given a decision maker's willingness to pay for each additional QALY.

### Sample size calculation

A total of 350 participants (175 per group) will be recruited. The study will have 80% power to detect as significant at the 5% level a 30% reduction in the rate of falling (i.e. an IRR of 0.70 using negative binomial regression analysis) in the 12-month follow-up period. This number will also be sufficient to detect clinically- and statistically-significant 10% between-group differences in lower extremity Summary Performance Scores and the self-reported mobility-related disability measure (power = 80%, p = 0.05, dropouts = 15%). A previous study among community dwellers found a 12% between-group difference on a similar measure of independence in activities of daily living.[36]

## Discussion

There is mounting evidence that falls and disability can be minimized in older people with well-designed exercise interventions. It is likely that bigger absolute effects would be seen in those at increased risk of falls and disability. Targeting exercise intervention programs to people at increased risk without expensive large-scale screening programs would be difficult. People who have been in hospital are known to be at increased risk of falls and disability so we have developed an exercise program and recruitment strategy for use in this population.

It is likely that a program with a greater amount of contact from a health professional would have greater effects on falls and disability. However, a high-contact program would be harder to implement in routine care due to the limited resources of health systems internationally. Thus, we aimed to develop a program which is intense enough to be effective yet inexpensive enough to be widely implemented if shown to be effective. The economic analysis will establish costs and benefits of our program.

The WEBB exercise program which we are trialing has been based on many years of research and clinical experience into the prescription of exercise for older people. The program allows for flexibility in exercise prescription to best cater for the needs of this relatively heterogeneous population. Thus, this program is optimally designed to address the major problems of falls and disability in older adults and could be readily translated into clinical practice. The study we are undertaking is designed and adequately-powered to assess the effect of the program.

This study will determine the impact of this exercise intervention on mobility-related disability and falls in older people who have recently been in hospital. In addition this project will identify of predictors of adherence with the program and will involve an economic analysis. There will be far-reaching benefits for older people, their carers and the community if this program can be demonstrated to assist in improving the level of physical functioning and reduce the number of falls in this high-risk population.

## Competing interests

The authors declare that they have no competing interests.

## Authors' contributions

This manuscript was drafted by CS and SDO. All authors are actively involved in the study. Most authors contributed to the writing of the grant application for this project that was funded by the Australian National Health and Medical Research Council in 2007. The grant application formed the basis for this manuscript. All authors contributed to the manuscript's critical review and approved the final version.

## Pre-publication history

The pre-publication history for this paper can be accessed here:



## References

[B1] Li R, Iadarola A, Maisano C (2007). Why Population Aging Matters: A Global Perspective.

[B2] World Health Organisation (2002).

[B3] Australian Institute of Health and Welfare (2008). Australia's Health 2008.

[B4] Pahor M, Blair SN, Espeland M, Fielding R, Gill TM, Guralnik JM, Hadley EC, King AC, Kritchevsky SB, Maraldi C (2006). Effects of a physical activity intervention on measures of physical performance: Results of the lifestyle interventions and independence for Elders Pilot (LIFE-P) study. J Gerontol A Biol Sci Med Sci.

[B5] Lord SR, Ward JA, Williams P, Anstey KJ (1993). An epidemiological study of falls in older community-dwelling women: the Randwick falls and fractures study. Aust J Public Health.

[B6] Mahoney J, Sagar M, Dunham NC, Johnson J (1994). Risk of falls after hospital discharge. J Am Geriatr Soc.

[B7] Gillespie LD, Gillespie WJ, Robertson MC, Lamb SE, Cumming RG, Rowe BH Interventions for preventing falls in elderly people (Cochrane Review). Cochrane Database Syst Rev.

[B8] Sherrington C, Whitney J, Lord S, Herbert R, Cumming R, Close J (2008). Effective exercise for the prevention of falls – a systematic review and meta-analysis. J Am Geriatr Soc.

[B9] Gill TM, Allore HG, Holford TR, Guo Z (2004). Hospitalization, restricted activity, and the development of disability among older persons. JAMA.

[B10] Hauer K, Rost B, Rutschle K, Opitz H, Specht N, Bartsch P, Oster P, Schlierf G (2001). Exercise training for rehabilitation and secondary prevention of falls in geriatric patients with a history of injurious falls. J Am Geriatr Soc.

[B11] Hauer K, Pfisterer M, Schuler M, Bartsch P, Oster P (2003). Two years later: a prospective long-term follow-up of a training intervention in geriatric patients with a history of severe falls. Arch Phys Med Rehabil.

[B12] Vogler C, Sherrington C, Ogle S, Lord S Reducing risk of falling in older people discharged from hospital: a randomised controlled trial comparing seated exercises, weight-bearing exercises and social visits. Arch Phys Med Rehabil.

[B13] Latham NK, Anderson CS, Lee A, Bennett DA, Moseley A, Cameron ID, Fitness Collaborative G (2003). A randomized, controlled trial of quadriceps resistance exercise and vitamin D in frail older people: the Frailty Interventions Trial in Elderly Subjects (FITNESS). J Am Geriatr Soc.

[B14] Tinetti ME, Williams CS (1997). Falls, injuries due to falls, and the risk of admission to a nursing home. New Eng J Med.

[B15] Marcus BH, Williams DM, Dubbert PM, Sallis JF, King AC, Yancey AK, Franklin BA, Buchner D, Daniels SR, Claytor RP (2006). Physical activity intervention studies: what we know and what we need to know: a scientific statement from the American Heart Association Council on Nutrition, Physical Activity, and Metabolism (Subcommittee on Physical Activity); Council on Cardiovascular Disease in the Young; and the Interdisciplinary Working Group on Quality of Care and Outcomes Research. Circulation.

[B16] Stiggelbout M, Hopman-Rock M, Crone M, Lechner L, van Mechelen W (2006). Predicting older adults' maintenance in exercise participation using an integrated social psychological model. Health Educ Res.

[B17] Ashworth NL, Chad KE, Harrison EL, Reeder BA, Marshall SC (2005). Home versus center based physical activity programs in older adults. Cochrane Database Syst Rev.

[B18] Folstein MF, Folstein SE, McHugh PR (1975). "Mini-Mental state": a practical method for grading the cognitive status of patients for the clinician. J Psychiatr Res.

[B19] Canning C, Sherrington C, Lord S, Fung V, Close J, Latt M, Howard K, Allen N, O'Rourke S, Murray S (2009). Exercise therapy for prevention of falls in people with Parkinson's disease: A protocol for a randomised controlled trial and economic evaluation. BMC Neurology.

[B20] Sherrington C, Lord S, Herbert R (2003). A randomised trial of weight-bearing versus non-weight-bearing exercise for improving physical ability in inpatients after hip fracture. Aust J Physiother.

[B21] Sherrington C, Lord SR, Herbert RD (2004). A randomized controlled trial of weight-bearing versus non-weight-bearing exercise for improving physical ability after usual care for hip fracture. Arch Phys Med Rehabil.

[B22] Olivetti L, Schurr K, Sherrington C, Wallbank G, Pamphlett P, Kwan MM-S, Herbert RD (2007). A novel weight-bearing strengthening program during rehabilitation of older people is feasible and improves standing up more than a non-weight-bearing strengthening program: a randomised trial. Aust J Physiother.

[B23] Marcus BH, Banspach SW, Lefebvre RC, Rossi JS, Carleton RA, Abrams DB (1992). Using the stages of change model to increase the adoption of physical activity among community participants. Am J Health Prom.

[B24] Haley SM, Andres PL, Coster WJ, Kosinski M, Ni P, Jette AM (2004). Short-form activity measure for post-acute care. Arch Phys Med Rehabil.

[B25] Onder G, Penninx BW, Ferrucci L, Fried LP, Guralnik JM, Pahor M (2005). Measures of physical performance and risk for progressive and catastrophic disability: results from the Women's Health and Aging Study. J Gerontol A Biol Sci Med Sci.

[B26] Guralnik JM, Simonsick EM, Ferrucci L, Glynn RJ, Berkman LF, Blazer DG, Scherr PA, Wallace RB (1994). A short physical performance battery assessing lower extremity function: association with self-reported disability and prediction of mortality and nursing home admission. J Gerontol.

[B27] Gibson MJ, Andres RO, Isaacs B, Radebaugh T, Worm-Petersen J (1987). The prevention of falls in later life. Dan Med Bull.

[B28] Lord SR, Menz HB, Tiedemann A (2003). A physiological profile approach to falls risk assessment and prevention. Phys Ther.

[B29] Lord SR, Fitzpatrick RC (2001). Choice stepping reaction time: A composite measure of falls risk in older people. J Gerontol A Biol Sci Med Sci.

[B30] Barnett A, Smith B, Lord SR, Williams M, Baumand A (2003). Community-based group exercise improves balance and reduces falls in at-risk older people: a randomised controlled trial. Age Ageing.

[B31] Moseley AM, Adams R (1991). Measurement of passive ankle dorsiflexion: Procedure and reliability. Aust J Physiother.

[B32] Brooks R, Rabin R, de Charro F (2003). The measurement and valuation of health status using EQ-5D: A European perspective.

[B33] Kempen GI, Yardley L, van Haastregt JC, Zijlstra GA, Beyer N, Hauer K, Todd C, Kempen GIJM, Yardley L, van Haastregt JCM (2008). The Short FES-I: a shortened version of the falls efficacy scale-international to assess fear of falling. Age Ageing.

[B34] Robertson MC, Campbell AJ, Herbison P (2005). Statistical analysis of efficacy in falls prevention trials. J Gerontol A Biol Sci Med Sci.

[B35] Perera S, Mody SH, Woodman RC, Studenski SA, Perera S, Mody SH, Woodman RC, Studenski SA (2006). Meaningful change and responsiveness in common physical performance measures in older adults. J Am Geriatr Soc.

[B36] Gill TM, Baker DI, Gottschalk M, Peduzzi PN, Allore H, Van Ness PH (2004). A prehabilitation program for the prevention of functional decline: effect on higher-level physical function. Arch Phys Med Rehabil.

